# Prevalence of *Helicobacter pylori* in Children and Their Family Members in a District in Turkey

**Published:** 2007-12

**Authors:** Abdullah Ceylan, Ercan Kırımi, Oğuz Tuncer, Kürşat Türkdoğan, Sevil Arıyuca, Nesrin Ceylan

**Affiliations:** 1 Department of Pediatrics; 2 Department of Gastroenterology, Faculty of Medicine, Yüzüncü Yil University, Van, Turkey

**Keywords:** Gastritis, *Helicobacter* infections, *Helicobacter pylori*, Turkey

## Abstract

The aim of this study was to determine the prevalence of *Helicobacter pylori* among children and their family members and to evaluate some epidemiologic characteristics. The study included 275 children, aged 1-15 year(s), suffering from different gastrointestinal complaints. Blood serology and stool antigen testing were used for the diagnosis of infection due to *H. pylori*. Sixty-five (23.6%) of the 275 children were positive for *H. pylori*, and this positivity had a significantly increasing correlation with age (p<0.001). *H. pylori*-associated infection was observed among 45 (69.2%) and 17 (8%) mothers in the *H. pylori*-infected and non-infected groups respectively (p<0.0001). Most children and their families infected with *H. pylori* were living in an urban area. The findings suggest that infection due to *H. pylori* is a problem for this district area, and all children having any gastrointestinal complaints should be examined whether *H. pylori* was prevalent among their family members.

## INTRODUCTION

*Helicobacter pylori* is a spiral gram-negative microorganism that is distributed worldwide. It is estimated that over 50% of the world population are infected with *H. pylori* (1–5). *H. pylori*–associated infection is either usually clinically silent or its signs and symptoms are non-specific. Gastroesophageal reflux, esophagitis, delayed gastric emptying, and various motilitiy disorders can be a sign or symptom of it (1–5). However, these symptoms are seen in many childhood illnesses. Younger children with peptic complaints may not have symptoms as clear as those of older children, and diagnosis of infection due to *H. pylori* is more difficult (6–10).

The epidemiology of *H. pylori*-associated infection is variable, since the prevalence is significantly higher and infection occurs in earlier ages in developing or poor countries compared to developed countries (11). In Turkey, the seroprevalence of infection due to *H. pylori* in children has been reported as high as 43.9% and 53.9% (3,6,7). There is an obvious public-health problem of *H. pylori*-associated infection, and thus, to design targeted, cost-effective prevention strategies, elucidation of the mode of transmission for this infection is crucial. Infection due to *H. pylori* is typically acquired in early childhood and usually persists throughout life unless specific treatment is given (12).

Definitive routes of its transmission have not yet been characterized, and the principal reservoir appears to be family members. Person-to-person transmission via the faecal-oral and oral-oral routes have been proposed. Numerous studies also indicate that low socioeconomic status, including domestic overcrowding, is a major risk factor for a higher prevalence of infection (13,14).

Currently, little data exist regarding the epidemiology of *H. pylori*-associated infection in our region. Therefore, this study was conducted to determine the prevalence of *H. pylori* among children and also to investigate if there is an association between *H. pylori* positivity and symptoms of children and their family members in a district in Turkey.

## MATERIALS AND METHODS

In total, 275 children with different gastrointestinal complaints, admitted to the Department of Pediatrics, Faculty of Medicine, Yüzüncü Yil University, were examined for *H. pylori* during May 2003–March 2005. Patients who were aged less than one year or aged over 15 years or who had any other chronic diseases or used medicines which affect the gastrointestinal system were excluded. All children and their families were questioned about gastrointestinal symptoms or complaints, e.g. recurrent abdominal pain, nausea, vomiting, gastroesophageal reflux, dyspeptic complaints. Cases who had both positive serology and positive stool antigen test were accepted as positive for infection due to *H. pylori*.

Both blood sera and stool specimens were studied in all children and their families. Three mL of venous blood were taken from all children and family members, and those sera were stored at −20 °C in a deepfreeze. After adequate sera were collected, antibody IgG against *H. pylori* was detected by the ELISA method. For this procedure (General Biological Corp-GBC No: 6 Innovatien First Road Science Based Ind Park Hsinchu, Taiwan), test-kits were used. The values above the cut-off level were accepted as *H. pylori*-positive and values below the cut-off level as *H. pylori*-negative. Hp faecal antigen (Premier *H. pylori*, Meridian Diagnostic Inc.) tests were performed as a diagnostic panel in all cases.

All patients were asked for information on gastrointestinal symptoms and place of residence (urban or rural) if there are other members of the family who had gastrointestinal complaints.

Statistical analysis was made using chi-square test analysis by the SPSS software (version 11.5). A value of p<0.05 was accepted as significant.

Written informed consents were taken from families for further investigation and analysis. The ethics committee of the hospital approved the study.

## RESULTS

In total, 275 children—140 boys (50.9%) and 135 (49.1%) girls aged 1-15 year(s) (mean±SD age 8.2±3.4 years) were enrolled in the study. Group 1 consisted of 28 children (10.18%) aged 1-5 year(s), group 2 consisted of 116 children (42.18%) aged 6-10 years, and group 3 consisted of 131 children (47.63%) aged 11-15 years. Sixty-five infected children were also divided into three age-groups. When these groups were compared for *H. pylori* positivity, each group was significantly (p<0.0001) different from any other group. *H. pylori* positivity had a significantly increasing correlation with age (Table 1).

**Table 1:** Age distribution of *H. pylori*-infected and non-infected children

		Infected (n=65)		Non-infected (n=210)	
Group	Age-group (years)	No.	%	No.	%

Group 1	1-5	3	4.62	25	11.90
Group 2	6-10	16	24.62	100	47.62
Group 3	11-15	46	70.76	85	40.48

p<0.0001 for trend with age

Gastrointestinal symptoms were similar among both *H. pylori*-infected and non-infected children. Some diagnoses of non-infected children were as follows: parasitic infection, peptic ulcer, gastritis, and familial mediterranean fever. Table 2 presents symptoms of all children. Families of all children were evaluated for *H. pylori*. The prevalence of infection was observed among members of the corresponding family: 45 (69.2%) mothers, 27 (41.5%) fathers, and 17 (26.1%) siblings in the infected children group. The prevalence of *H. pylori* was higher among children with a family member infected with the bacterium than among children without any infected family member. The differences were significant for mothers, fathers, and also siblings (p<0.0001, Table 3).

**Table 2:** Gastrointestinal symptoms of children included to study

	*H. pylori*-infected		Non-infected	
Symptom	No.	%	No.	%

Recurrent abdominal pain	49	75.3	150	71.4
Nausea	32	49.2	95	45.2
Vomiting	24	36.9	45	21.4
Gastroesophageal refux	15	23.0	45	21.4
Burning pain in the abdomen	28	43.0	63	30.0
Haematemesis	3	4.6	14	6.6

**Table 3:** Distribution of family members for *H. pylori* positivity

Family member	Infected children (n=65)	Non-infected children (n=210)	p value

Mother			
Yes	45	37	<0.0001
No	20	173	
Father			
Yes	27	22	<0.0001
No	48	203	

Of the 275 children studied, 236 (85.8%) were living in an urban area and 39 (14.2%) in a rural area. According to *H. pylori*-positive results, most (93.8%) children were living in an urban area, and only four (6.2%) were living in a rural area. When the prevalence of infection was compared [61 (25.8%) of 236 children in an urban area and 4 (10.26%) of 39 children in a rural area], the results were significant (p<0.05) (Table 4).

**Table 4:** Distribution of children according to place of residence

Gender	*H. pylori*-infected (n=65)	Non-infected (n=210)

Urban	61	175
Rural	4	35

## DISCUSSION

The prevelance of *H. pylori* varies from country to country and by age-groups. In the United States, *H. pylori* positivity in children aged less than 10 years is lower than 5%, whereas it is 10% and 60% among individuals who are aged 20 years and above 60 years respectively (8). In Japan, the prevalence of *H. pylori*-associated infection was 29% among persons aged 15-19 years (15). The prevelance of *H. pylori* was 34% among children, aged less than 12 years, in Italy (9). In Bangladesh, Mahalanabis *et al.* studied 469 children, aged 6-9 years, with urea breath test and found the prevalence of *H. pylori*-associated infection in 84% of them (16). In Australia, Mitchell *et al.* reported the prevalence of *H. pylori*-associated infection of 14.1% among 227 children, aged 6 months-18 years, who had endoscopy due to gastrointestinal symptoms (17). In the present study, we found a moderate result (23.6%) for *H. pylori*-positive infection among children aged 1-15 year(s). Investigators from Turkey reported the seropositivity of *H. pylori* in 43.9% of 346 children aged 6 months-16 years and in 64.4% of 466 children aged 6-17 years (18,19). Us and Hasçelik studied 657 asymptomatic patients from various age-groups about *H. pylori* positivity, of whom 348 patients (53.9%) were positive (7). These results suggest that different results can be found with respect to the prevalence of *H. pylori* in the same country even in geogrpahically close provinces.

*H. pylori*-associated infection can be observed in early childhood. In developing and poor countries in particular, children with *H. pylori* suffer at lower ages than in developed countries. Environmental factors of children, such as education level of parents, number of siblings, and economic factors are important in *H. pylori*-associated infection (20–22). Investigators have reported an increasing prevalence of *H. pylori*-associated infection with an increasing age in asymptomatic people around the world (23–31). Mitchell *et al.* found positive serology by the age of five years in 23% of children in southern China (26). The present study has also shown a significant increase in the prevalence of *H. pylori*-associated infection with an increasing age (p<0.001).

Anyone, including a child, can have an infection due to *H. pylori* without any symptoms. When the bacterium causes symptoms, they are usually have either symptoms of gastritis or peptic ulcer disease. In children, symptoms of gastritis may cause nausea, vomiting, and frequent complaints about pain in the abdomen, although these symptoms are seen in many childhood illnesses (6,7). Some investigators reported some data about signs and symptoms of *H. pylori*-associated infection in children. Biswal *et al.* reported *H. pylori* positivity at a rate of 65.4% in patients who had recurrent abdominal pain (32). Yağcı *et al.* conducted a study with 143 *H. pylori*-positive (determined by endoscopy) patients, 102 (71%) of them admitted to the hospital with chronic abdominal pain, 23 (16%) with haematemesis and melaena, and seven (5%) with vomiting (3). In our study, we had observed recurrent abdominal pain in 49 (75.3%), nausea in 32 (49.2%), vomiting in 24 (36.9%), gastroesophageal reflux in 15 (23%), and haematemesis in three (4.6%) children infected with *H. pylori*.

Some authors have demonstrated intrafamilial clustering of *H. pylori*-associated infection (33). Drumm *et al.* found positive serology in more than 80% of siblings of children colonized with *H. pylori* compared to 13% of age-matched controls (34). Malaty *et al.* also provided data demonstrating an increased prevalence of colonized children of infected parents (35). The higher prevalence of infection due to *H. pylori* in parents of infected children suggests person-to-person transmission within the family (36,37). In the present study, we observed rates of higher positivity among children of infected parents compared to other children whose parents were not infected. An infected mother may play a key role in the transmission of *H. pylori* within the family (38). Mouth secretions of mother which may be contaminated with *H. pylori* may be transmitted to the infant and child (39,40). Transmission may occur also for using common spoons, the licking of pacifiers, or teats of feeding bottles, or even for chewing or tasting children's food (38).

Person-to-person transmission of *H. pylori* may be an important mode of disease spread, but the rate of transmission is still a controversial issue (33). However, faecal-oral transmission may play an important role in developing countries (38,41). Transmission of the microorganism may be facilitated due to lack of hygiene, for crowding of people, and through more intimate contact. In addition to the environmental factors contributing to transmission, results of a study suggest that host factors may be important in pathogenesis (43). Boren *et al.* reported the involvement of Lewis blood group antigen in the attachment of *H. pylori* to gastric mucosa (42). It was also reported that the HLA DQAI gene contributes to the host response against *H. pylori* (43). In the present study, we demonstrated a very strong association between the infection status of children and of their mothers.

Findings similar to those in our study, reporting an overall higher rate of infection in an urban population (25.8%) than in a rural population (10.2%), have been reported from Nepal and Viet Nam (44,45), although their rates of infections were higher than ours. Also, a study in Mexico reported that the urban people had the high risk for seropositivity (46).

In conclusion, the present study reports a significant increase in the seroprevalence of infection due to *H. pylori* with increasing age. It also provides strong evidence for a transmission pathway from family members to children. Urban children have a greater risk for infection due to *H. pylori*.
